# Antiplatelet protocol: Effects of ingesting a tomato pomace extract on human platelet aggregation

**DOI:** 10.1016/j.mex.2019.08.012

**Published:** 2019-08-24

**Authors:** Aníbal Concha-Meyer, Eduardo Fuentes, Iván Palomo

**Affiliations:** a Centro de Estudios en Alimentos Procesados (CEAP), CONICYT-Regional, Gore Maule, R09I2001, Talca, Chile; b Facultad de Ciencias Agrarias, Universidad de Talca, Talca, Chile; c Thrombosis Research Center, Medical Technology School, Department of Clinical Biochemistry and Immunohaematology, Faculty of Health Sciences, Interdisciplinary Center on Aging, Universidad de Talca, Talca, Chile

**Keywords:** Antiplatelet, Human platelet, Aggregation, Tomato pomace

## Abstract

Consumption of tomato and tomato-based products is associated with risk reduction of cardiovascular disease (CVD). Protective effect of tomato is not clearly understood, but there is an interest in its ability to affect platelet aggregation (coagulation). Therefore, the aim of the present study is to describe the protocol of a single blind, parallel design (3-groups), placebo controlled trial, which will assess acute and “acute upon 5-day repeated dose” effects of ingesting a tomato pomace extract on platelet aggregation. Participants will be randomized to receive a flavoured water either with 1 g tomato pomace extract, 2.5 g tomato pomace extract or placebo negative control. A total of 99 people are required to complete (n = 33/group). Each group will ingest either a treatment or placebo once daily, for 5-days and blood samples will be taken to analyze platelet aggregation. For 14-days preceding the baseline assessment day and for the 5-day period of intervention, participants will have to exclude some foods/beverages from their diet, which are known to affect platelet function. The present study is expected to generate significant information about the effect of tomato pomace extract consumption on platelet function of volunteers.

## Graphical abstract

**Figure:**
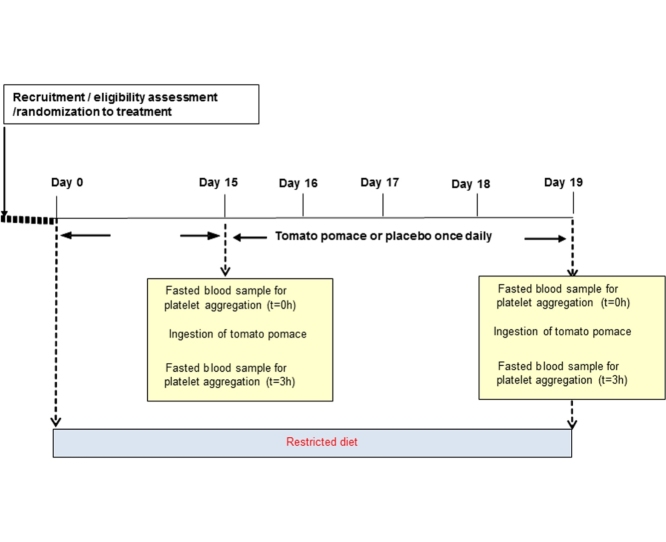

**Specifications Table**

**Table:** 

Subject Area:	*Pharmacology, Toxicology and Pharmaceutical Science*
More specific subject area:	*Nutrition*
Protocol name:	*Antiplatelet protocol*
Reagents/tools:	*NA.*
*Experimental design:	*Single blind, parallel design and placebo controlled trial.*
Trial registration:	*Clinicaltrials.gov (Ref: NCT02986165)*
Ethics:	*The trial will be conducted in the research facility at the University of Talca and Centro de Estudios en Alimentos Procesados (CEAP), furthermore all procedures will be approved by the University of Talca Research Ethics Committee. Each participant will give written informed consent prior to taking part in the trial.*

*Value of the Protocol

**Table:** 

• *Protocol of platelet aggregation in human ex vivo.* • *A rapid cost-effective method to evaluate the antiplatelet activity of natural products.*

## Description of protocol

Evidence from human intervention trials and mechanistic studies suggests that tomato and tomato based products are associated with a reduction in CVD risk [[1], [2], [3], [4]]. The mechanism by which this protective effect occurs is not clearly understood, but research has focused on its potential to modulate platelet function [[5], [6], [7]]. In a single-blind, randomized, parallel design human intervention trial (3-groups), we aim to recruit 99 participants (33/group) to ingest an orange flavoured beverage containing different doses of a tomato pomace extract (1 and 2.5 g,) or placebo control over a 5-day period. The acute and “acute upon 5-day repeated dose” effects of the treatments on platelet aggregation will be evaluated at the start and end of the intervention period. Before implementing this large scale human intervention trial we will also conduct a preliminary ‘ascending dose human tolerance study’ in a small group of participants across a ˜3 week period. Ascending dose of tomato pomace extract will be 1, 2.5 and 10 g based on preliminary work of platelet aggregation inhibition in animal model and food matrix formulation assays [2,8,9].

### Study hypothesis

Daily ingestion of a tomato pomace extract for 5 days will significantly reduce platelet aggregation in humans compared with a placebo control and in a dose dependent manner.

### Study objectives

1) To determine the acute effect of ingesting two different doses of a tomato pomace extract on platelet aggregation
2) To determine the “acute upon 5-day repeated dose” effect of ingesting two different doses of a tomato pomace extract on platelet aggregation

### Study design

**Fig. 1:**
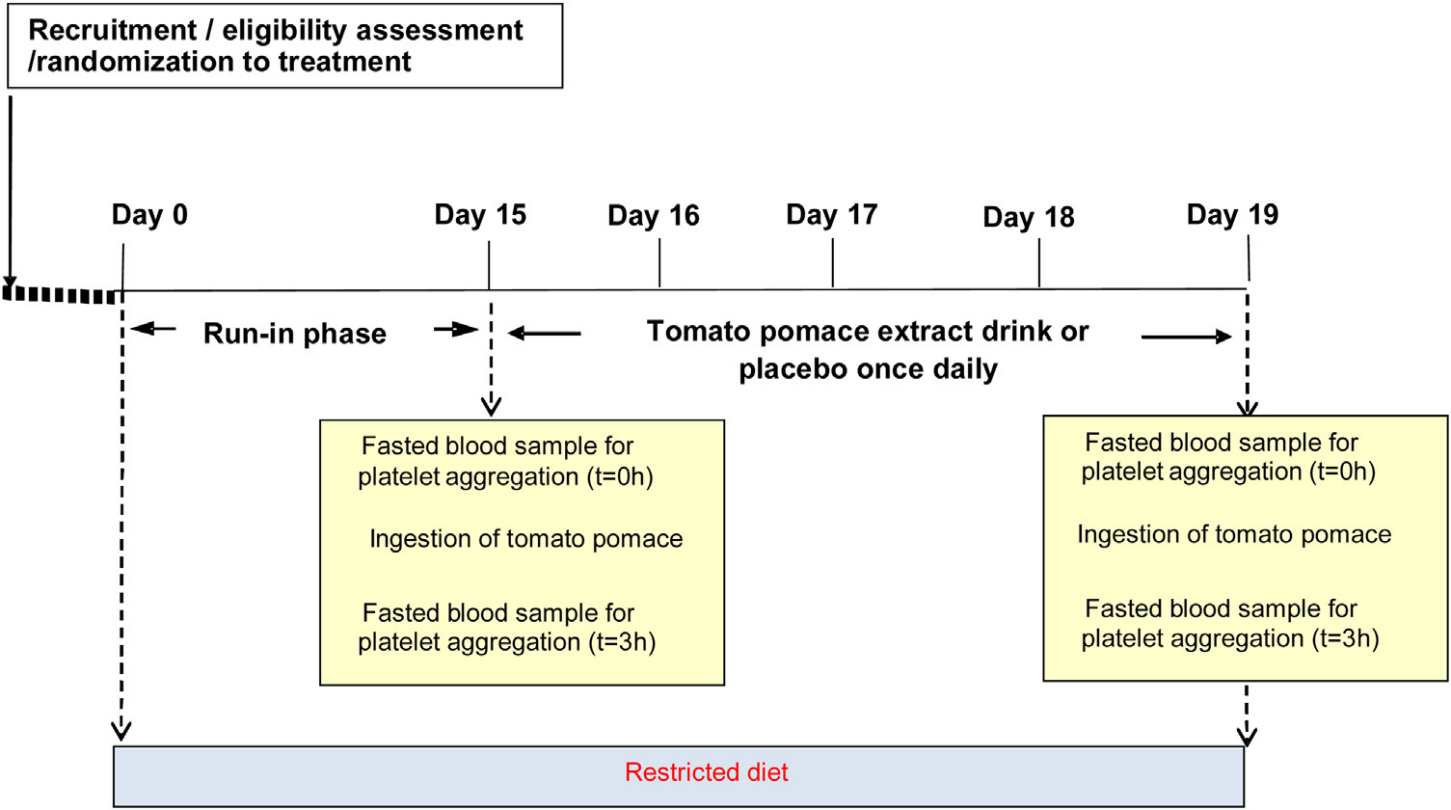
Schematic overview of antiplatelet study design.

A single blind, parallel design (3-groups), placebo controlled trial will be conducted to assess the acute and “acute upon 5-day repeated dose” effects of ingesting a tomato pomace extract on platelet aggregation. Participants will be randomized to receive either: a) 1 g tomato pomace extract delivered in flavoured water b) 2.5 g tomato pomace extract delivered in flavoured water or c) Flavoured water (placebo control). A total of 99 people are required to complete study (n = 33/group), which was calculated for an effect size of 17.4%, standard deviation within group of 19.5% and significance alpha level of 0.05 for 90% power, following O’Kennedy et al. [10] information. Each group will ingest either a treatment or placebo once daily, for 5-days. For 14-days preceding the baseline assessment day and for the 5-day period of intervention, participants will completely exclude from the diet some foods/beverages that are known to affect platelet function (tomato and all tomato based products, alcohol, cocoa and cocoa products and green tea). To assess the acute effects of the intervention on platelet aggregation, a blood sample will be obtained before ingestion (D15; 0-h) and post ingestion (D15; 3-h) of the treatment. Given the half-life of platelets (approximately 9–11 days), the objective was to evaluate the effect of continuous doses for 5 days on the same pool of circulating platelets. To assess the “acute upon 5-day repeated dose” effects, further blood samples will be taken at the end of the intervention period (D19; 0 and 3-h). Fig. 1 shows a schematic overview of the study design.

### Inclusion criteria

• Healthy men aged between 18 and 26 years
• BMI > 19.5 and <26.0 kg/m^2^
• Platelet aggregation response corresponding to ≥60%

### Exclusion criteria

• Dietary supplements and tomato allergy
• Chronic diseases and active treatment (e.g. cancer, diabetes, asthma, and cardiovascular and gastro-intestinal diseases)
• Smokers
• Medication known to affect platelet function (e.g. aspirin, clopidogrel and non-steroidal anti-inflammatory drugs, among others)
• Alterations of hemostasis

### Recruitment strategy

The study population will include apparently healthy men aged 18–26 years. Only males will be selected to assure a lower variability, since other studies have demonstrated sex-related variations in platelet count which are higher in women [11]. Furthermore, age related changes in platelet function, such as interactions with von Willebrand Factor, are higher in women than in men [12]. A total of 99 participants are required to complete this trial. However, recruitment for this study will only cease once we are confident that the required number of participants will complete the study.

Recruitment of participants to this trial will be achieved through poster and e-mail advertisements placed across the University of Talca, inviting anyone who is interested in receiving information about the study to contact the named researchers. Those expressing an interest will be provided with a participant information sheet (annex 1). If insufficient numbers of people are recruited from the University of Talca we may place advertisements in the local media.

After reading the information sheet, those interested in taking part will be invited to the research facility for an informal meeting with a member of the research team, who will explain the study focusing on the participant’s involvement. Participants will be encouraged to ask questions at this stage prior to making any commitment. At the end of the meeting, participants will be given ample opportunity to consider whether they wish to take part in the study. If after the consideration period they wish to participate in the study, they will be asked to contact a member of the research team to arrange an appointment at the research facility for the pre-study clinical screening.

### Clinical screening

Participants will attend research facilities after 10 h of fasting over night, but they will be advised to drink water during the fasting period. Upon arrival at the research facility a member of the research team will review the consent form and answer any participant’s questions that may arise at this stage. Consent will have to be signed to participate in the study. A copy of this form will be given to the participant to save. A nurse will then be in charge of completing a basic health assessment questionnaire, in addition to measuring and recording blood pressure, pulse, height and weight and calculating the body mass index. Clinical screening for this trial will be conducted taking 10 ml blood sample to perform biochemical profile and as platelet aggregation response assay [13]. The biochemical profile will include: glucose, urea nitrogen, urea, total cholesterol, uric acid, total proteins, albumin, globulins, total bilirubin, GOT transaminase, GPT transaminase, GGT, lactate dehydrogenase, alkaline phosphatase, total calcium and phosphorus. The results of the blood count and biochemical profile will be reviewed by a medical doctor and members of the research team. In the event that the doctor considers that the results of the blood test indicate a health problem (which may compromise the well-being of the participant in case he takes part), then participant will be excluded from the study and will be properly informed. Those participants who meet the basic inclusion / exclusion criteria will be eligible to participate in this study.

### Participant randomization

Participants in this parallel design study will ingest 1 or 2.5 g tomato pomace extract incorporated into a drink or a placebo control drink, over a 5 day period. Each treatment will be assigned a letter (A–C). Every participant that will be recruited, will be designated with the next allocated treatment. Randomization to treatment will be assigned by the computer programme randomization.com. Participants will be blinded to the treatments.

### Preparation of tomato pomace drinks

Immediately prior ingestion, ‘drinks’ containing the appropriate mass of tomato pomace extract will be prepared at the research facility as follows:

1) Low dose pomace extract – 1 g powder will be accurately weighed before dissolving in flavoured water to a final mass of 100 g
2) High dose pomace extract – 2.5 g powder will be accurately weighed before dissolving in flavoured water to a final mass of 100 g
3) Placebo control – 100 g flavoured water

Orange flavoured water is necessary to ensure that the participants are blinded to the test product consumed.

### Intervention procedure

The test arm will comprise a 5 day intervention period which will be preceded by a run-in phase of 14 days. For standardisation purposes all trial procedures between days 15–19 will be conducted in the morning time following an overnight fast. A test arm is described below.

Days 1–14 – During the 14-day ‘run-in’ period, participants will be instructed to avoid foods and beverages known to significantly contribute to anti-platelet activity. A 14-day period is thought to be sufficient as this comprises the average lifespan of platelets (˜10 days) and allows for equilibration to dietary change. The foods and beverages to be avoided include tomato and all tomato based products, alcohol, cocoa and cocoa based products and green tea, which are known to contain a significant concentration of compounds that affect platelet function such as ethanol, flavonoids, polyphenols, carotenoids and nucleosides, among others. In the event that a participant self-prescribes medication for minor illness during this time period, he will be instructed to contact a member of the research team who will assess the impact of the medication on platelet function and decide whether or not the run-in period should be re-scheduled.

Day 15 – Participants will arrive at the research facility on the morning of day 15 having fasted from 10 pm the night before. Prior to conducting any clinical procedures the participant will be asked some basic health related questions (Annex 2) to establish whether or not any changes in medical conditions/medications etc. have occurred since their last visit and that they are well enough to proceed with the study day assessment. A baseline (t = 0 h) fasted blood sample (15 mL) will be collected by venepuncture. Once collected, participants will ingest the allocated treatment drink. A further blood sample will be obtained 3-h post ingestion (15 mL) to assess the acute effects of tomato pomace ingestion on platelet aggregation. At the end of the assessment period participants will be free to eat and drink ad libitum, however they must continue avoiding foods and beverages known to significantly contribute to platelet activity (tomato and all tomato based products, alcohol, cocoa and cocoa based products and green tea).

Days 16–18 – Participants will return to the research facility each morning having fasted from 10 pm the night before. The allocated treatment will be prepared fresh each morning by a member of the research team and ingested by the participant whilst in the research facility. On departure, the participants will be instructed to continue avoiding the foods and beverages known to significantly contribute to platelet activity.

Day 19 – Participants will return to the research facility on the morning of day 19 having fasted from 10 pm the night before. Prior to conducting any clinical procedures the participant will again be asked some basic health related questions (Annex 2) for the reasons already described. A blood sample (15 mL) will be collected by venepuncture. The participant will once again ingest the allocated treatment drink and a further fasted blood sample collected 3-h later. This completes participation in the trial.

### Treatment compliance

Each participant will ingest the allocated treatment at the research facility.

### Assessment of platelet function

Whole blood will be collected by venepuncture directly into 3.2% sodium citrate tubes (9:1 v/v) after discarding the first 2.0 mL of the draw. Tubes will be centrifuged at 240 x g for 10 min. to obtain platelet-rich plasma (PRP). After aliquoting a portion of the PRP for platelet count (hematologic counter) the tubes will be further centrifuged at 650 x g for 10 min. to obtain platelet-poor plasma (PPP). The PRP will be adjusted to 200 × 10^9^ platelets/ L with the PPP [14].

Aliquots of the adjusted PRP (480 μL) will be pre-incubated (37 °C; 3 min) before the addition of the platelet agonists ADP (final conc. 2 and 4 μmol/L) and collagen (final conc. 0.5 and 1 μg/mL). Platelet aggregation will be measured by light transmission according to Born and Cross using a Lumi-aggregometer [15]. The result of platelet aggregation (% maximal amplitude) will be determined using the software AGGRO/LINK.

### Power calculations & statistical analysis

The primary outcome measure for this study is platelet aggregation. The sample size has been powered on data from a previous study reported by O’Kennedy et al. [10], which is a study not too dissimilar to this one. In this trial, the effect size in platelet response between treatment (18 g tomato extract syrup) and control after stimulation of platelets with collagen was 17.4% with a standard deviation of 19.5%. Assuming a similar maximum difference between the control and the 2.5 g dose of tomato pomace extract, and half this difference for the 1 g dose of tomato pomace extract then to gain sufficient evidence to reject the null hypothesis of no differences between the 3 groups at the 5% significance level with 90% power would require 33 participants per group to complete the trial. The data will be analysed using one-way between subjects analysis of variance or its non-parametric equivalent (Kruskal–Wallis test by ranks). Post hoc comparisons between groups will use Tukey’s honest significant difference (HSD) multiple comparison procedure.

### Reporting of adverse event/reactions and serious adverse events

Adverse events are defined as any undesirable experience occurring to a subject during the study, whether or not considered related to the tomato pomace extract. An adverse reaction is defined as all noxious and unintended responses to the tomato pomace extract related to any of the doses tested. The phrase responses to the tomato pomace extract means that a causal relationship between the product and an adverse event is at least a reasonable possibility, i.e. the relationship cannot be ruled out. All adverse events and reactions reported by the subject or observed by staff will be recorded (Annex 3). A serious adverse event (SAE) is defined as any untoward medical occurrence or effect that at any dose results in death, is life threatening (at the time of the event), requires hospitalization or results in persistent or significant disability or incapacity. SAE’s must be reported by the CI immediately to the University of Talca ethics committee and the study sponsor (CEAP). In this instance the trial will be immediately suspended or terminated.

### Informed consent

All participants will give written informed consent before they can participate in the trial. Informed consent will be obtained before any study specific procedures are conducted. It will be made clear to each participant that he is free to withdraw his consent at any time and without reason.

### Participant fasting

This study requires participants to be fasted on assessment days. To reduce the length of the fasting period study measurements will be conducted in the morning. Participants will be able to drink ad libitum during the fasting period. At clinical screening, a blood sample will be collected for blood glucose analysis to exclude undiagnosed diabetic participants.

### Confidentiality

Participants will be assigned a unique code which will be used to anonymize all biological samples and data arising from this trial. Anonymized information/data will be kept in locked cabinets at the research facility and processed and stored on a computer system.

All personal information will be kept confidential and known only to the Chief Investigator and study personnel at the University of Talca. Personal information will be kept separately to coded information in locked cabinets at the research facility for a period of 5 years.
